# Microfluidic Devices for Terahertz Spectroscopy of Live Cells Toward Lab-on-a-Chip Applications

**DOI:** 10.3390/s16040476

**Published:** 2016-04-04

**Authors:** Qi Tang, Min Liang, Yi Lu, Pak Kin Wong, Gerald J. Wilmink, Donna D. Zhang, Hao Xin

**Affiliations:** 1 Department of Electrical and Computer Engineering, University of Arizona, Tucson, AZ 85721, USA; tangqi@email.arizona.edu (Q.T.); minliang@email.arizona.edu (M.L.); 2 Department of Biomedical Engineering, Pennsylvania State University, University Park, PA 16802, USA; luyi@psu.edu (Y.L.); pak@engr.psu.edu (P.K.W.); 3 WiseWear Corporation, New York, NY 10018, USA; jerry.wilmink@gmail.com; 4 College of Pharmacy, University of Arizona, Tucson, AZ 85721, USA; dzhang@pharmacy.arizona.edu

**Keywords:** cell trapping, lab-on-a-chip, microfluidics, THz spectroscopy

## Abstract

THz spectroscopy is an emerging technique for studying the dynamics and interactions of cells and biomolecules, but many practical challenges still remain in experimental studies. We present a prototype of simple and inexpensive cell-trapping microfluidic chip for THz spectroscopic study of live cells. Cells are transported, trapped and concentrated into the THz exposure region by applying an AC bias signal while the chip maintains a steady temperature at 37 °C by resistive heating. We conduct some preliminary experiments on *E. coli* and T-cell solution and compare the transmission spectra of empty channels, channels filled with aqueous media only, and channels filled with aqueous media with un-concentrated and concentrated cells.

## 1. Introduction

In recent years, biological and medical applications of THz technologies have developed rapidly, e.g., in cancer diagnosis, body imaging, and biological spectroscopy [1,2]. Studies show that the low-frequency collective vibrational modes of many large biomolecules (e.g., proteins and DNAs) and biological cells have a time scale on the order of picoseconds, which corresponds to the THz frequency range, *i.e.*, 0.1 THz to 10 THz [3,4,5,6,7,8,9]. Therefore, THz spectroscopy may become a powerful label-free and non-invasive tool for studying the structure and behavior of a wide range of biological systems from molecular to organism levels. However, the experimental study of live cells in aqueous media is still a challenge mainly due to the large absorption of water, the lack of proper THz sources, and the sample preparation difficulties.

The aqueous environment in which cells live has an important effect on both THz absorption and biological function. However, biological samples in earlier studies of THz spectroscopy are usually dehydrated due to the considerable absorption of water at THz frequencies [3,4]. Such dehydrated samples would lead to low vibrational mode intensity and poor reproducibility. Most works have underscored the importance of developing novel microfluidic sample holders to improve the measurement reproducibility and efficiency [5,6,7,8,9,10,11,12,13].

Microfluidics is a powerful technique used in the analysis of biological particles within an extremely small volume of liquid. A microscale channel can avoid excessive water absorption by the aqueous environment, thus enabling the spectroscopy measurement of live cells in aqueous media at THz frequencies. Microfluidics can also manipulate bioparticles for *in situ* sample preparation [14,15,16,17,18]. Biological samples, such as DNA, large proteins and individual cells, can be concentrated or trapped within a small region of the channel.

Microfluidic devices have been applied in liquid characterization and bio-sensing at microwave frequencies [19,20,21,22]. The dielectric properties of liquid mixtures and cell solutions are characterized by measuring either the shift of resonant frequency or the waveguide impedance. In addition to microwave spectrum, THz band is very intriguing because of the existence of low-frequency vibrational modes from biomolecule-solvent dynamics and interactions, which play an important role in biological functions [3,4,5,6,7,8,9]. Some efforts have been reported on using microfluidic devices at THz frequencies [10,11,12,13], however, mostly for sensing liquid mixtures [11,12,13]. George, *et al.* proposed a PDMS-Zeonor microfluidic device and measured THz absorption spectra of bovine serum albumin [10].

In this study, we establish a simple and cost-effective microfluidic chip which can provide efficient and reliable measurement of THz spectra of live cell samples. We observe that different cells are concentrated at different locations close to the electrodes. The underlying trapping mechanism is explained by positive or negative dielectrophoretic force [15]. We show that the temperature of the channel can be controlled and maintained at 37 °C by resistive heating. Two sets of THz systems are used for the spectral measurement, including a time-domain pulsed system and a frequency-domain continuous wave system. Preliminary experiments of *E. coli* bacteria and T-cell solutions are conducted and the results are reported.

This paper is organized as follows: Section 2 presents the chip configuration and performance, including the cell-concentration capability and the temperature distribution. Section 3 discusses the experimental setup and test procedure for the THz spectroscopic measurement. Section 4 presents the experimental results of *E. coli* and T-cell solutions, and discusses potential future directions. Section 5 summarizes the paper.

## 2. Microfluidic Chip

### 2.1. Chip Design and Fabrication

**Figure 1:**
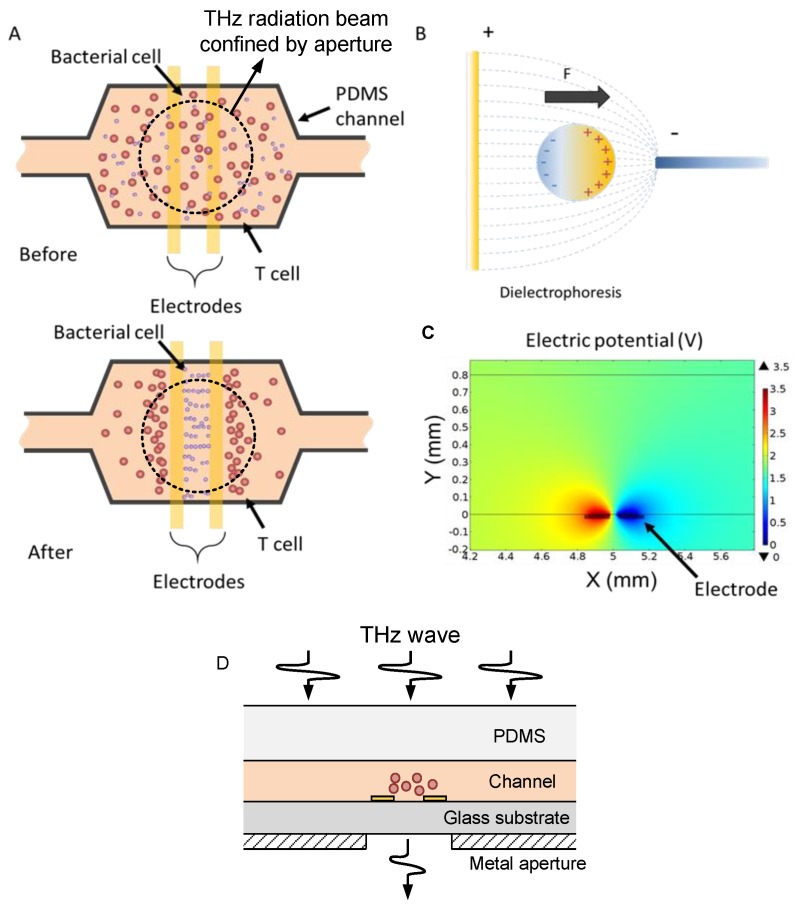
(**a**) The schematic top view of the microfluidic chip: before and after applying voltage on the electrodes; (**b**) Principle of dielectrophoresis; (**c**) Simulation result: electric potential distribution from the cross-sectional view of the microfluidic chip; (**d**) The schematic cross-sectional view of the chip configuration.

A schematic view of the microfluidic chip is shown in Figure 1a,d. The device consists of a glass substrate, a pair of parallel gold electrodes for dielectrophoretic cell manipulation, and a bonded PDMS microchannel. The pair of electrodes are deposited onto the quartz substrate by lift-off. The PDMS channel is then fabricated by soft lithography. PDMS is the most common elastomeric material used in microfluidic devices for biological applications because of its tunable surface properties, transparency to visible light, non-toxicity to cells, and ease of fabrication. The dielectric property of PDMS is firstly characterized at THz frequencies. The relative dielectric constant is about 2.5 and the loss tangent is about 0.05 around 0.3 THz. The major concern in the design of microfluidic devices is minimizing the absorption and multireflection loss from the device in the THz spectral measurement. Channel thickness is a key parameter to reduce the loss of aqueous media. Channel width is 5 mm. In this study the channel thickness is 300 μm, which is small enough for reducing absorption loss and large enough for cells to pass. The thickness of PDMS layer should be as thin as possible while maintaining its mechanical strength. In this study, the PDMS layer thickness is reduced to 1.44 mm. The width of the electrode is 100 μm, and the gap distance between the electrodes is 50 μm.

### 2.2. Cell Trapping

DEP arises from the interaction between a non-uniform electric field and the induced dipole of a polarizable object (Figure 1b). The dielectrophoretic force can transport the object toward the high electric field region or low electric field region depending on the effective polarization between the object and the medium. If the object has a higher polarizability, the force will push the object toward the high electric field strength region (positive DEP); otherwise, the force will point toward the low electric field strength region (negative DEP). An electric potential distribution of the chip is shown in Figure 1c. DEP has been demonstrated to effectively manipulate various types of biomolecules, particles and cells. The time-averaged dielectrophoretic force on a spherical object is given by [17]:

(1) $$F_{DEP}=2\pi R^{3}\varepsilon_{m}Re\left\{K\left(\omega \right)\right\}\nabla {\left|E_{rms}\right|}^{2}$$

where *R* is the particle radius, *E*_rms_ is the root mean square electric field, ω is the angular frequency, and *K*(ω) is the Clausius-Mossotti factor, which describes the frequency variation of the effective polarizability of the particle in the medium. The Clausius-Mossotti factor is defined by:

(2) $$K\left(\omega \right)=\frac{\varepsilon_{p}^{*}-\varepsilon_{m}^{*}}{\varepsilon_{p}^{*}+2\varepsilon_{m}^{*}}$$

where $\varepsilon_{p}^{*}$ and $\varepsilon_{m}^{*}$ are the complex permittivities of the particle and medium, respectively. For a homogenous material, the complex permittivity is given by:

(3) $$\varepsilon^{*}=\varepsilon +\frac{\sigma }{j\omega }$$

where ε is the permittivity and σ is the conductivity of the particle and medium.

**Figure 2:**
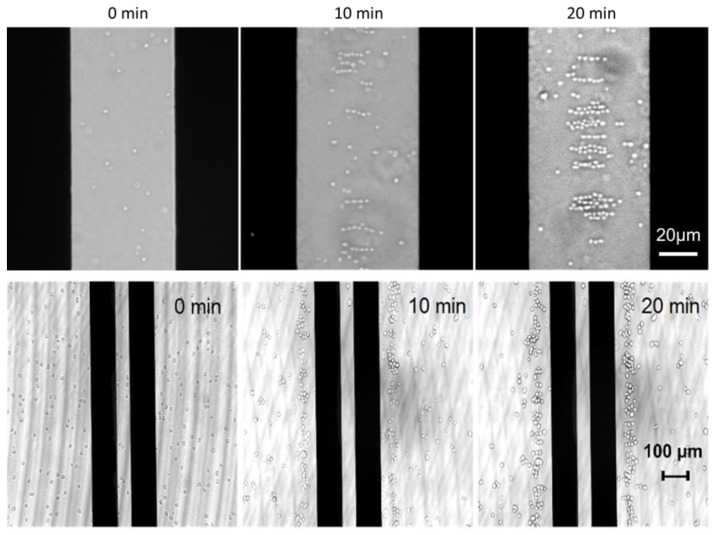
A microscopic view of cell concentration process as a function of time: (Upper row) *E. coli* bacteria (bright dots) are concentrated between the inner edges of the electrodes (black area), and (Bottom row) T-cells (bright dots) are concentrated near the outer edges of the electrodes (black strips).

In the experiment, the cell solution was pipetted into the microfluidic channel. Firstly cells distributed evenly in the microchannel before a voltage was applied to the parallel electrodes. Then a 1 MHz square wave AC signal with a peak-to-peak voltage (V_pp_) up to 7V was applied across the electrodes. In our observation, cells moved toward the center of the channel and aggregated near the electrodes within a few minutes (Figure 1(a)). Figure 2 shows the bright field image of low-density *E. coli* bacteria in Luria Bertani (LB) medium and T-cells in RPMI medium (4.7 × 10^3^ cells/mL). The *E. coli* bacteria were concentrated inside the parallel electrodes due to positive DEP. In contrast, T-cells were pushed away from the electrodes by negative DEP. This ability potentially allows cell separation and selective detection of cells in different regions of the channel. The separation is primarily due to the intrinsic difference in dielectrophoretic responses of the cells, which have different Clausius-Mossotti factors [18].

### 2.3. Thermal Distribution

The temperature of the cell solution is another important consideration in biological experiments. Either too high or too low a temperature would lead to the inactivation of cells or biomolecule analytes. This is a problem especially for long-term experiments, e.g., studies of THz- induced biological effects and spectroscopy that need large numbers of sample averages. Taking the assessment of DNA damage as an example, the operation time can last from 1 to 24 hours depending on different requirements [2]. An incubator is normally used for maintaining a constant temperature.

**Figure 3:**
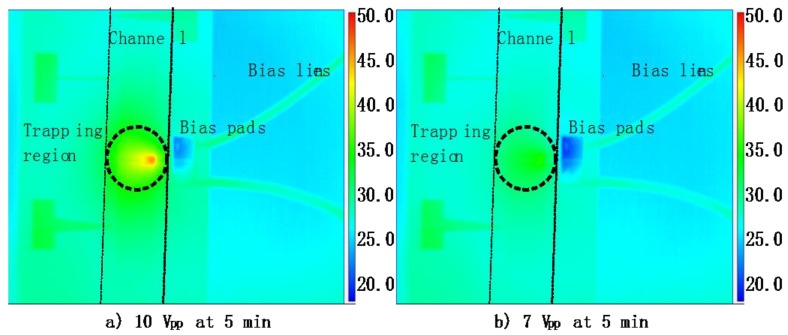
Thermal images of the microfluidic chip at different bias voltage with the solution conductivity of 1.36 S/m (captured by a FLIR 6000 IR camera).

**Figure 4:**
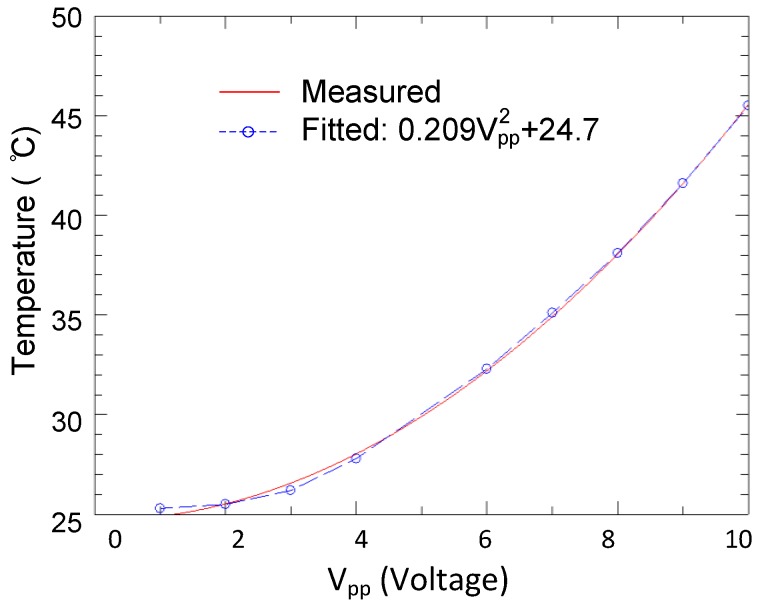
The maximum temperature in the channel as a function of the peak to peak voltage.

For our microfluidic chip, the AC bias of the chip generates a dielectrophoretic force inside the channel, and also leads to a Joule heating-induced temperature increase. Fortunately, the temperature of the cell solution can be controlled steadily by applying an appropriate bias voltage in a solution with known conductivity. Figure 3 presents infrared thermometry results of the microfluidic device filled with the cell solution under different bias conditions. The device shows a rather uniform temperature distribution near 35 °C with 7 V, which is close to the physiological temperature. For a 10 V bias case, the temperature in the region of concentration can increase to over 50 °C. Figure 4 plots the maximum temperature in the channel as a function of the peak to peak voltage. A curving fitting is applied and plotted in the inset of Figure 4. The maximum temperature near the electrodes increases as a quadratical function of the bias voltage, as expected.

## 3. THz Spectroscopy of the Live Cell Microfluidic Chip

Two different THz measurement systems are used in this work, *i.e.*, a pulsed TDS system and a frequency-domain CW amplified multiplier chain system. In the TDS system, THz pulses are emitted and detected using near-infrared femtosecond laser pulses by a coherent and time-gated method [23]. It has advantages of high temporal resolution (broadband), fast response, and high SNR. It is very efficient in spectral measurement due to its simultaneous acquisition of signals in a broad bandwidth. However, the TDS system usually has a limited spectral resolution (usually ~10 GHz) because of the trade-off between the spectral resolution Δ*f* and the temporal measurement window T (Δ*f*=1/T). The maximum duration of the temporal window is limited by the repetition rates of the laser source, the length of the scanning delay, and fundamentally the noise level in the system [24]. The output power of TDS system is also limited at the level of μW. The frequency-domain CW system based on amplified multipliers has higher spectral resolution and larger transmitted power compared to the TDS system. However, the CW system takes much longer time in the spectroscopy measurement, and the bandwidth is usually limited for a certain system setup.

In this work, we applied both THz measurement systems. A TDS system (T-Ray 2000 from Picometrix, Ann Arbor, MI, USA) was firstly used for measuring the *E. coli* samples. The spectral resolution is 10 GHz. Secondly, an amplifier multiplier chain based CW system (VDI-AMC-S156, from Virginia Diodes, Inc., Charlottesville, VA, USA) was used for the measurement of the T-cell samples. The spectral resolution is 1 GHz. The bandwidth is from 0.14 to 0.22 THz. The output power is about 0.5 to 3.5 mW. 

### 3.1. Configuration

**Figure 5:**
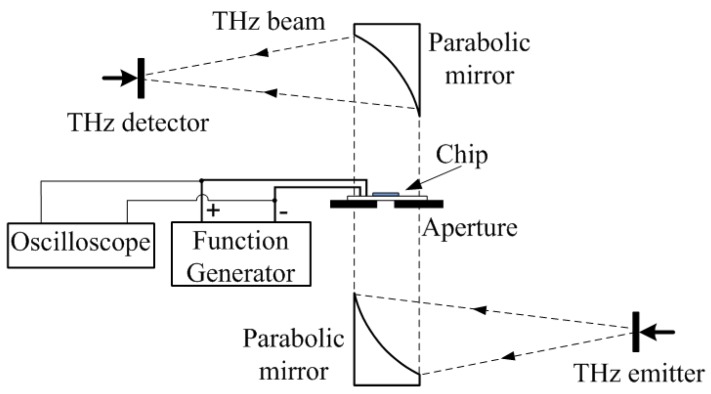
The diagram of the experimental configuration of the THz measurement system.

Figure 5 illustrates the details of the experimental setup. A perpendicular optical path is configured so that the microfluidic chip can be placed horizontally. The beam width of the THz wave is about 1 cm. A sample holder with a small aperture is used to secure the chip. The aperture size is 4 mm in diameter so that the THz radiation is confined within the channel.

### 3.2. Measurement Procedure

**Figure 6:**
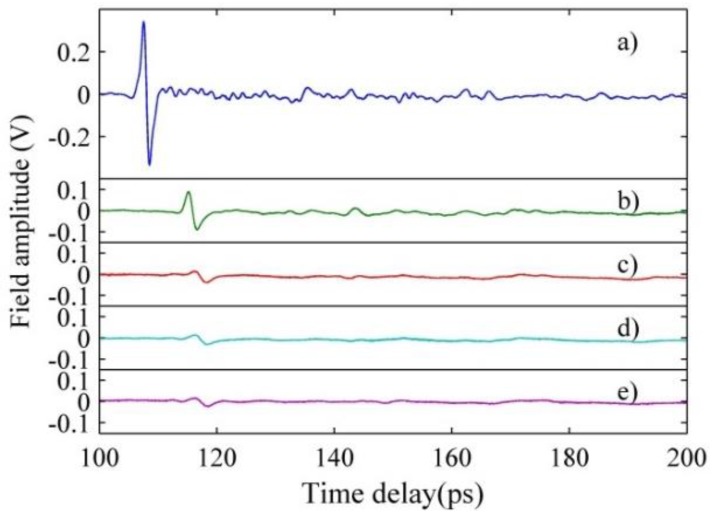
Measured THz time-domain signals of *E. coli* bacteria in LB medium (8.3 × 10^8^ CFU/mL) for different cases: (**a**) Aperture; (**b**) Empty chip; (**c**) LB medium; (**d**) V_off_; and (**e**) V_on_.

The measurement procedure is given as follows: (1) measure the transmission spectra of the aperture as a reference; (2) measure the spectra of an empty chip on the holder; (3) pipette the LB medium for the *E. coli* bacteria measurement or the RPMI medium for the T-cell measurement (without any cells) into the chip and measure the spectra; (4) inject T-cell solutions and measure the spectra without the AC bias being applied; (5) turn on the AC bias and wait for certain period of time until the cells are concentrated as shown in Figure 2; (6) measure the spectra of cell-trapping case. For each experiment, we repeat the same procedure with independent setup for three times to reduce manual alignment errors and system uncertainties. The averaged measurement results for the above sequential experiments will be presented as: (a) the aperture only (aperture), (b) the empty chip with the aperture (empty chip), (c) the chip filled with medium without any cells (medium), (d) the chip filled with cell solution without biasing (V_off_), and (e) the chip filled with concentrated cell solution (V_on_).

## 4. Results

### 4.1. E. coli Solution

**Figure 7:**
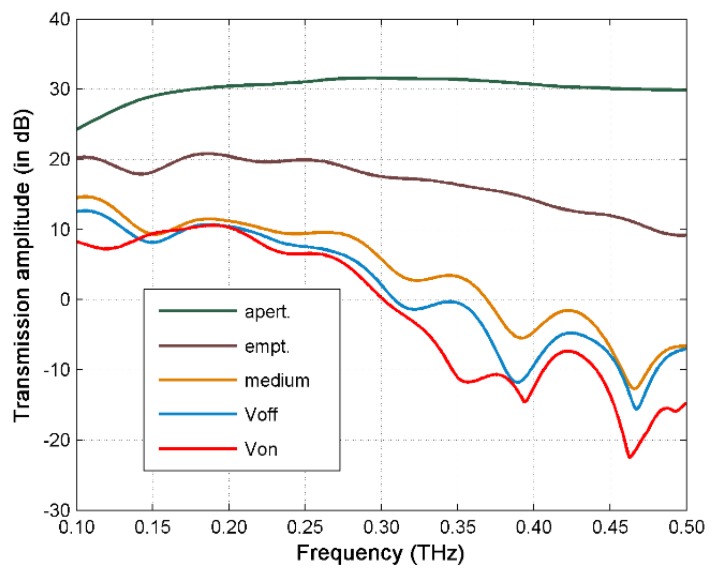
The FFT spectra of the measured THz time-domain signals of *E. coli* bacteria in LB medium (8.3 × 10^−8^ CFU/mL) with 7 V bias voltage. Note that the cell density in this experiment is much larger than the trapping experiment shown in Figure 2.

Figure 6 shows the measured time-domain signals of *E. coli* in LB medium with the TDS system. Figure 7 plots the corresponding spectra obtained by Fast-Fourier Transforms (FFT) with a 30 ps truncation of the temporal window to reduce the influence from multireflections. The noise level is about −30 dB, which is measured by blocking the detector and turning off the THz source.

From Figure 7, we can see that the empty chip results in a 4–20 dB loss of transmission, mainly due to PDMS absorption, multi-reflections and scatterings from the microfluidic chip. The aqueous medium in the 300 µm channel leads to a few (<10) dB loss at low frequency region and 10–22 dB loss above 0.2 THz. It can be observed that the transmission loss for the V_off_ cell solution case is consistently higher than that of the medium only case, indicating the THz response due to the un-concentrated cells. Moreover, the transmission loss for the V_on_ cell solution case is mostly higher than that of the V_off_ cell solution case, indicating higher THz absorption by the concentrated cells.

**Figure 8:**
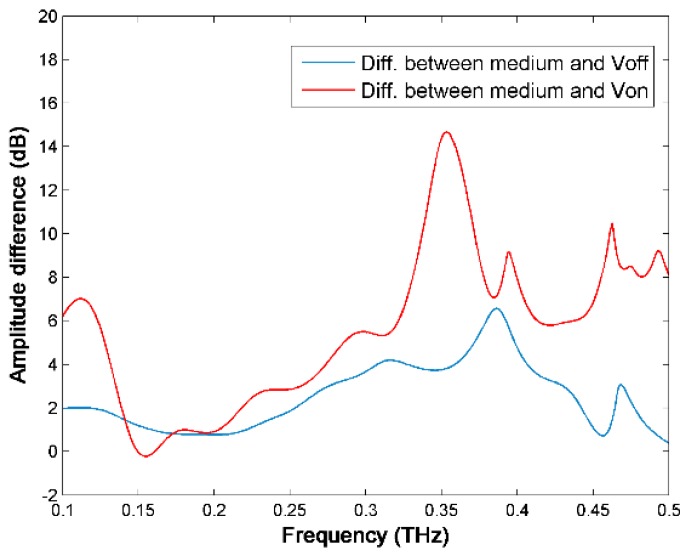
The spectral difference between the medium and V_on_ case and between the medium and V_off_ case.

In particular, we observe a 2 dB absorption increase near 0.11 THz, and similarly a 7 dB absorption peak in the spectral difference between the medium and Von case (see Figure 8) at the same frequency. There are also some transmission peaks at higher frequencies for both curves in Figure 7, especially for the concentrated cells (Von case). However, due to the relatively low signal to noise ratio, it is not clear whether those are THz resonances from the cells.

### 4.2. T-cell Solution

**Figure 9:**
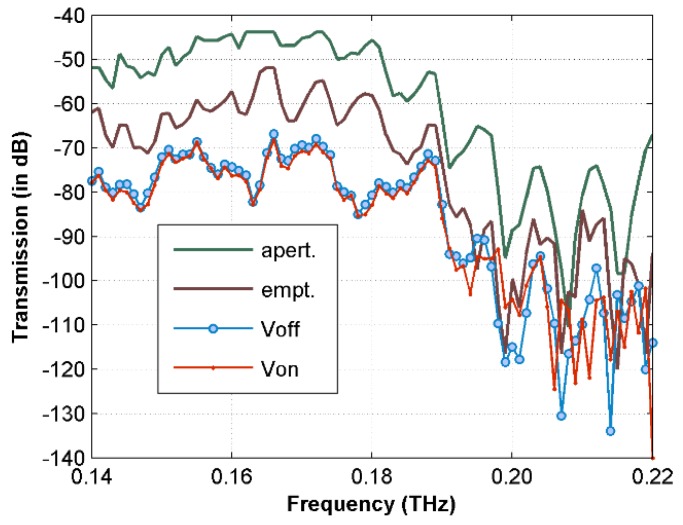
The measured transmission spectra of T-cells in RPMI medium (4.7 × 10^3^ cells/mL) using CW systems.

**Figure 10:**
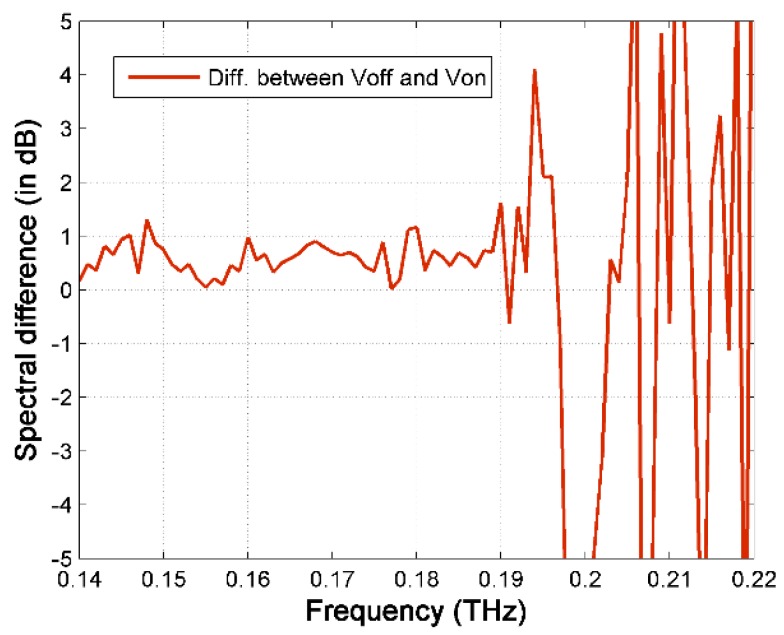
The spectral difference between the un-concentrated and concentrated T-cell sample.

Figure 9 presents the measured transmission spectra of T-cells in RPMI medium (4.7 × 10^3^ cells/mL) using the CW system. The cell solution case has about 10 dB higher THz transmission loss compared to the empty channel case. Figure 10 plots the spectral difference between the case of V_on_ and V_off_. The average transmission of the concentrated T-cell sample is still slightly lower up to 0.19 THz before the signal to noise ratio becomes much worse. Comparing to the *E. coli* bacteria case, the spectral difference between the un-concentrated and concentrated T-cell samples is much smaller. One of the reasons is that the trapping position for T-cells is at the outer edges of the electrodes (as shown in Figure 2) where the THz field intensity is smaller than that at the inner gap. In addition, the cell density of the T-cell sample is much smaller than that of the *E. coli* sample. It might also be possible that the intrinsic THz properties of T-cells are close to the RPMI medium, although further investigation is necessary to be conclusive.

### 4.3. Chip Optimization Discussion

The output power of THz source is usually limited. From Figure 7 and Figure 9, we can see that there is still large loss of the signal due to multi-reflections, scatterings and material absorptions from the microfluidic chip itself. The majority loss still comes from the aqueous media which limits the discriminative capability of such device. The presented 300 µm microfluidic channel already shows the advantage of reducing the absorption at THz frequencies. Chips with 800 and 600 µm channel thickness were also tested, but no detectable output signals were measured due to the large absorption of the excessive aqueous media. On the other hand, the channel thickness should be large enough for cell passing.

There are other solutions to further improve the SNR and to extend the spectral measurement to higher frequencies, e.g., by reducing the substrate thickness or utilizing low refraction index material for the substrate [25]. It can also help increase the signal intensity by using dielectric lens instead of using the aperture to focus the THz beam. THz sources, waveguides, and field enhancement structures can also be integrated to the chip to increase the interactions between cells and THz waves [11,26].

## 5. Conclusions

The work in this paper demonstrates an initial proof-of-concept for cell concentration, steady temperature control, and THz spectral measurement of live cells. The DEP-based cell manipulation capability has been successfully demonstrated. The microfluidic chip also provides the desired steady controllable temperature environment. Both time-domain and frequency-domain THz spectroscopy systems are used, each having its own advantages. Our experimental results on empty channels, channels filled with aqueous media only, and channels filled with un-concentrated and concentrated cell solutions show different THz transmission responses. In general, the concentrated cell samples are more absorptive than the un-concentrated case. An absorption peak is observed near 0.11 THz for both the un-concentrated and concentrated *E. coli* bacteria sample, which might indicate an absorption signature of *E. coli* bacteria. No absorptive signatures are observed for T-cell case. The ultimate goal of this work is to develop lab-on-a-chip devices at THz frequencies integrating functions including sample preparation, bio-particle transportation and concentration, and effective THz bio-sensing and spectroscopic study. This work not only shows encouraging results but also helps identify improvements needed, including the optimization of the chip design and THz source.

## Abbreviations

The following abbreviations are used in this manuscript:

CW: Continuous-wave
DEP: Dielectrophoresis
*E. coli*: Escherichia coli
LB: Luria Bertani
PDMS: Polydimethylsiloxane
RPMI: Roswell Park Memorial Institute
SNR: Signal-to-noise ratio
THz: Terahertz
TDS: Time-domain spectroscopy
